# Dogs can sense weak thermal radiation

**DOI:** 10.1038/s41598-020-60439-y

**Published:** 2020-02-28

**Authors:** Anna Bálint, Attila Andics, Márta Gácsi, Anna Gábor, Kálmán Czeibert, Chelsey M. Luce, Ádám Miklósi, Ronald H. H. Kröger

**Affiliations:** 1 Lund University, Department of Biology, Mammalian Rhinarium Group, Sölvegatan 35, 22362 Lund, Sweden; 2 MTA-ELTE Comparative Ethology Research Group, 1117 Budapest, Hungary; 3 Eötvös Loránd University, Department of Ethology, 1117 Budapest, Hungary; 4 MTA-ELTE “Lendület” Neuroethology of Communication Research Group, Hungarian Academy of Sciences - Eötvös Loránd University, 1117 Budapest, Hungary; 5 University of Bremen, Department of Ecology and Evolutionary Biology, Leobener Str., 28359 Bremen, Germany

**Keywords:** Sensory processing, Animal behaviour

## Abstract

The dog rhinarium (naked and often moist skin on the nose-tip) is prominent and richly innervated, suggesting a sensory function. Compared to nose-tips of herbivorous artio- and perissodactyla, carnivoran rhinaria are considerably colder. We hypothesized that this coldness makes the dog rhinarium particularly sensitive to radiating heat. We trained three dogs to distinguish between two distant objects based on radiating heat; the *neutral* object was about ambient temperature, the *warm* object was about the same surface temperature as a furry mammal. In addition, we employed functional magnetic resonance imaging on 13 awake dogs, comparing the responses to heat stimuli of about the same temperatures as in the behavioural experiment. The *warm* stimulus elicited increased neural response in the left somatosensory association cortex. Our results demonstrate a hitherto undiscovered sensory modality in a carnivoran species.

## Introduction

A conspicuous feature of most mammals is the glabrous skin on the nose-tip around the nostrils, called a rhinarium^1^. In moles (Talpidae) in general and in the star-nosed mole (*Condylura cristata*) in particular, the rhinarium has exquisite tactile sensitivity, mediated by a special sensory structure in the skin, Eimer’s organ^2^. In the raccoon (*Procyon lotor*) and the coati (*Nasua nasua*), two carnivoran species with well-developed rhinaria without Eimer’s organs, activity was elicited in the trigeminal ganglion by stimulation of the rhinarium skin with various non-chemical stimulus modalities^3^. The authors concluded that the rhinaria of the studied species seem to have a primary function other than gathering tactile information. Curiously, the temperature of the carnivoran rhinarium in awake animals is considerably lower than in other mammalian groups^4^. In alert dogs (*Canis familiaris*), the temperature of the rhinarium follows a characteristic pattern. At 30 °C, it is about 5 °C colder than ambient temperature, about equal at 15 °C, and about 8 °C at 0 °C ambient temperature^5^. Rich innervation by the trigeminus nerve^6a^ suggests a sensory function.

**Figure 1:**
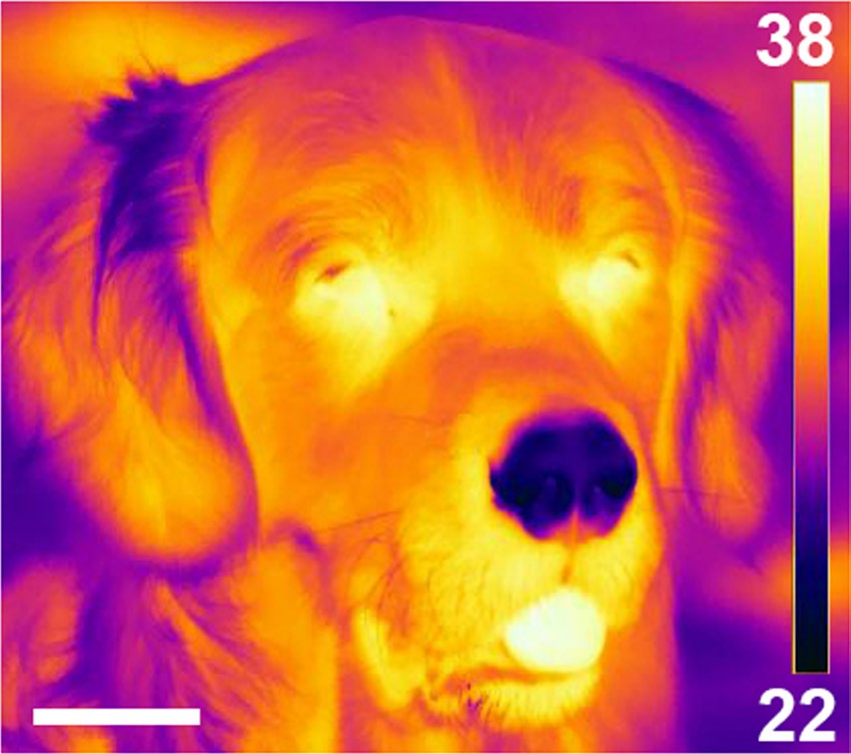
Thermograph of a dog in the shade at 27 °C ambient temperature. The colour scale on the right is in °C and can be used to read out approximate temperatures. Note the warm tongue and the cold rhinarium (hairless nose tip). Scale bar: 50 mm.

A role of the wet rhinarium in thermoregulation is unlikely, because its surface area is too small in relation to body size. Furthermore, if a dog is exposed to moderate heat stress and starts to pant, it extends the tongue from the open mouth (Fig. 1). The tongue is wet and warm, despite the airflow generated by panting, and is thus effectively dissipating surplus body heat by radiation and evaporation. The rhinarium, however, remains cold (Fig. 1) and is therefore ineffective.

Low tissue temperature affects metabolic functions in general and sensory sensitivity in particular (e.g.^7^), with one known exception: crotaline snakes cool their infrared-sensitive pit-organs by respiratory evaporation of water and strike more accurately with colder pit-organs^8^. Furthermore, colder snakes are more sensitive to thermal radiation^9^.

Detection of thermal radiation is challenging because of the low energy contents of long-wavelength photons. Ferrets (*Mustela putorius furo*) are able to see electromagnetic radiation of up to almost 1 µm in wavelength (near-infrared, NIR)^10^. A photon of thermal radiation in the middle-infrared (MIR, 3–5 µm) and far-infrared (FIR, >7 µm) bands cannot excite a photoreceptor because it has too little energy to isomerize a photopigment^11^. For biological detectors of thermal radiation, the only option is to detect the warming of the tissue by the absorption of many long-wavelength photons^12^.

The sensory membranes of snake pit-organs react to temperature changes as small as 0.001 degrees, possibly even smaller^13,14^. It is still unclear how the snakes transduce such tiny temperature differences to useful nervous signals because the molecular mechanism suggested^15^ cannot account for the performance of the snakes at temperatures below 25 °C^9^.

Crotaline snakes can strike at prey guided exclusively by the thermal radiation emanating from a warm body^16,17^. The closest wild relative of domestic dogs, the grey wolf (*Canis lupus*), preys predominantly on large endothermic prey e.g.^18^ and the ability to detect the radiation from warm bodies would be advantageous for such predators.

With this in mind, we designed two complementary series of experiments to test whether dogs can sense thermal radiation. We trained dogs on weak signals of thermal radiation at Lund University, Sweden, in a two-alternative forced-choice paradigm. At the Eötvös Loránd University in Budapest, Hungary, we performed functional magnetic resonance imaging (fMRI) experiments on awake dogs to elucidate where in the brain activity occurs if the animals detect a source of weak thermal radiation.

## Results

### Behavioural experiment

All three dogs could detect stimuli of weak thermal radiation in double-blind experiments (Table 1).

**Table 1:** Detection performances.

Dog	Choices (correct/total)	p-value
Kevin	32/40 (80%)	<0.001
Delfi	44/65 (68%)	0.003
Charlie	68/89 (76%)	<0.001

The performances of the three dogs in a two-alternative forced-choice experiment with thermal stimulation. Stimulus temperature was 11–13 °C higher than ambient temperature. The circular stimulus patches were 102 mm in diameter and had to be detected from a distance of at least 1.6 m, i.e. they subtended a solid angle of about 3.7 degrees. Their centres where at maximum 16 degrees apart. In all experiments, the dog handler did not know the answer and no other person could influence the dog.

### fMRI experiment

**Figure 2:**
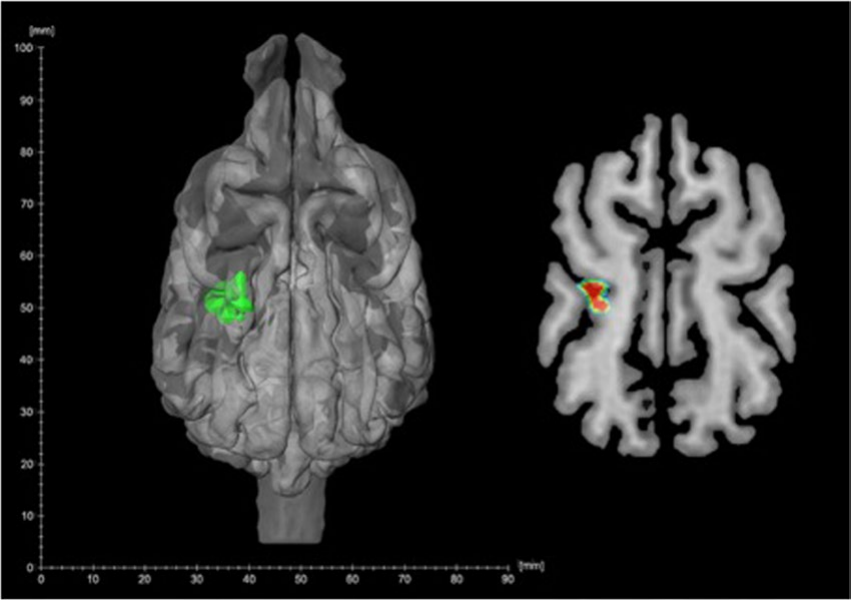
Significant cluster of 14 voxels in the left somatosensory association cortex. Single peak at x = −12, y = −14, z = 18 (T_(12)_ = 6.71, p < 0.001, p_FWE-corr_ = 0.038, k_E_ = 14). Left: shown on a reconstructed 3D image, Right: shown in a horizontal slice of the brain.

The whole-brain random effects analysis of the *warm* > *neutral* contrast revealed a significant cluster of 14 voxels (FWE-corrected at the cluster level) in the region of the left mid and rostral suprasylvian gyrus, with a single peak at x = −12, y = −14, z = 18 (T_(12)_ = 6.71, p < 0.001, p_FWE-corr_ = 0.038, k_*E*_ = 14). This cortical area is known as the somatosensory association cortex^6*b*,19,20^ (Fig. 2). We found no suprathreshold clusters in the right hemisphere and no brain regions were more responsive in the reversed, *neutral* > *warm* contrast. The lateralization analysis of the *warm* > *neutral* contrast revealed a significant left hemisphere bias (Mann-Whitney U test, U_(76)_ = 507, p = 0.011, two-tailed) in the symmetrical spherical volumes (r = 4 mm) around the peak voxel in the left and the right hemisphere.

## Discussion

The ability to sense weak thermal radiation has the potential of conveying valuable sensory information to an animal preying mainly on endothermic animals. The ability to sense such radiation is known in insects (Black fire beetle, *Melanophila acuminata*)^21^, reptiles (certain snake species: Crotalinae, Boidae)^22^ and one species of mammal so far, the common vampire bat, *Desmodus rotundus*), which can detect skin areas richly perfused with blood and thus suitable for biting after landing on a host animal^23^.

The rhinarium is the prime candidate as the sensory structure in dogs. Thermal photons are too weak to isomerize a photopigment in the eye^11^. Furthermore, the water in the aqueous and vitreous bodies of the eye effectively absorbs thermal radiation. Except for the rhinarium, all other parts of a dog’s face are covered by insulating fur. The sensitive area in vampire bats is also in the nasal region and it is also somewhat colder than other parts of the face^23^.

The cortical area showing significant activation in response to the *warm* condition is located at the caudal border of the left parietal somatosensory cortex^6*b*,19,20^. This area seems to play a crucial role in co-registering different sensory information (e.g. visual, somatosensory, proprioception) in order to plan and guide specific, goal-directed actions (e.g. targeting)^24^. This may suggest that the heat signal has been perceived as part of a complex environmental stimulus, eliciting the ‘neural planning’ of oriented, goal-directed actions. Assuming that a heat sense plays a role in predatory behaviour, this may also indicate a rapid integration of multisensory input and the subsequent motor output. Although the region of the cortical representation of the tip of the nose is only a few millimeters in size, given the location of the significant neural activation at the end of the rostral suprasylvian groove^19,20^ and the fact that the experiment was carefully controlled to preclude the activation of other sensory systems, we can state that the cortical region showing significant activation is most probably representing the nasal region. Also, the location of the detected activation is clearly distinguishable from the auditory and olfactory cortical areas. According to anatomical textbooks – based on lesion studies – the primary cortical olfactory area is the piriform lobe, as well as the lateral olfactory gyrus and the parahippocampal gyrus. Olfactory fibres originating from the olfactory bulb reach this area via the lateral olfactory tract, bypassing the thalamus along the way^19^. An fMRI study investigating the dog’s olfactory system in conscious and anesthetized animals found activation in the olfactory bulb and bilateral piriform lobes, including anterior olfactory cortex, piriform cortex, periamygdala, and entorhinal cortex for both low and high odour concentration^25^, while another found activation in the olfactory bulb, periamygdala, entorhinal cortex, and anterior cingulate cortex^26^. The primary auditory area of the dog lies largely in the temporal lobe, centred around the middle ectosylvian gyrus involving the caudal ectosylvian and sylvian gyri as well^6*b*,19^. The dorsal and rostral regions of the sylvian gyrus are part of the auditory association cortex^6*b*^. In two fMRI studies where the auditory regions were localized functionally, the following areas were found to be activated: (1) Sylvian gyri along the pseudosylvian fissure (extending ventrally toward the temporal pole) and the ectosylvian gyri along the ectosylvian sulcus extending dorsally to the suprasylvian sulcus^27^; (2) right middle suprasylvian sulcus; left middle ectosylvian sulcus; right caudal ectosylvian gyrus; left middle ectosylvian gyrus; right tectum mesencephali; left tectum mesencephali^28^. The above mentioned studies and anatomical data point to the fact that the heat stimulus was not detected by either the olfactory or auditory systems. Our finding of the left hemispheric bias of the cortical activation complements and lends further support to this assumption. In most studied vertebrates, feeding responses such as food discrimination or striking at prey were predominantly processed by the left hemisphere^29–33^, for broader reviews see^34–36^. In dogs specifically, it has been found that in a detour-task, attack-trained dogs that showed a preference using their right visual hemifield (mainly processed by the left hemisphere) needed less time to solve the task than those that preferably used their left visual hemifield, in line with the specialization of the left hemisphere in prey-catching behaviour^37^.

Our hemispheric asymmetry findings also seem to be in agreement with the Valence Model of cerebral emotional processing, since according to this hypothesis, positive emotions or approach-related emotional states – congruent with food-related neural responses – are processed predominantly in the left hemisphere^29,38–40^. Corresponding to this hemispherical lateralization, asymmetrical behaviours have been found in dogs in response to different types of emotional stimuli. For instance, dogs have been found to use their left nostril more often to sniff human odours collected during fearful situations and physical stress, suggesting a left hemisphere bias^41^. An interesting hypothesis regarding this lateralized behaviour is that these fearful heterospecific chemosignals elicit dogs’ prey drive reflected in left-lateralized neural activity^40^. In another instance, Quaranta *et al*. (2007) have found that dogs preferentially wagged their tail to the right (contralateral, left hemisphere control) when presented with positive stimuli (i.e. the dog’s owner), while the opposite direction was preferred with emotionally negative stimuli (i.e. a dominant dog)^42^. Similar results have been found in the study of Racca *et al*. (2012) where dogs presented with expressive dog faces showed a right gaze bias (left hemisphere advantage) while looking at positive facial expressions, but a left gaze bias (right hemisphere advantage) while looking at negative facial expressions^43^. A left hemisphere bias has also been shown in dogs for processing species-typical vocalizations, unless the sounds elicited intense emotions including fear^44^.

In the fMRI experiment, the presence or absence of a heat source had to be detected and we studied what part of the brain was activated when weak thermal radiation was sensed. In the behavioural experiments, the dogs had to locate a heat source. The total radiation from the stimulus-presenting apparatus was always the same and the dog had to determine on which side the *warm* stimulus was presented. Our results show that dogs can sense weak thermal radiation, use the sensory information for directed behaviour, and that the somatosensory system is activated by such radiation.

It is unclear how thermal radiation is transduced in the dog rhinarium. Its skin and underlying tissue is compact and devoid of cavities. The innervation consists of many large, heavily myelinated axons in the dermis, which rise up far into the epidermis close to the skin’s surface in numerous pegs or papillae of dermis tissue^6*c*,45^. In contrast, the pit-organs of crotaline snakes e.g.^13,46,47^ and the microbolometers in “simple” thermal cameras^48^ share the feature of a thin, lightweight structure that is readily warmed by impinging thermal radiation.

The goal of our experiments was to test the abilities of dogs in general. Determining thresholds at various ambient temperatures, with various rhinarium temperatures, for different stimulus sizes, distances, and temperatures requires further studies. In this investigation, we used dogs of various breeds and sizes as well as two different experimental approaches and found that sensing weak thermal radiation is within the abilities of the species *Canis familiaris*. The limits and mechanisms of this ability remain to be elucidated.

## Methods

### General issues

Accurate temperature measurements were essential in our studies for determining rhinarium, stimulus, and ambient temperatures. We used a FLIR E30 or a FLIR T640 thermographic camera (FLIR Systems, Wilsonville, USA), equipped with an 18.0 mm (E30) or a 24.6 mm (T640) lens. In the behavioural experiment, the FLIR E30 camera was used to take measurements of the dogs’ rhinaria and the stimuli, while the FLIR T640 camera was used to record the experimental sessions. In the fMRI experiment, the FLIR T640 camera was used to take measurements of the dogs’ rhinaria and the stimuli. The measurements were taken from a distance of 0.5 to 1.0 m. Temperature values from the thermographs were read out from the screen in the case of the FLIR E30 camera, while those taken by the FLIR T640 camera were evaluated with the FLIR Tools Plus software (FLIR Systems). In the latter case, the temperatures were determined as averages of pixel values in a manually selected area on the rhinarium as in^4,5^.

Warm and cold are relative terms and therefore some definitions are in order. In our work, *warm* means warmer than ambient temperature so that there was a temperature contrast. We call the cold stimulus *neutral* because its temperature was as close as possible to ambient temperature (thermoneutral, 13), i.e. there was no or only a very small such contrast.

All stimuli of radiating heat used in our experiments were too weak to be felt by human hands, even at very short distances. We had to touch the surfaces to feel the warmth.

### Behavioural experiment

#### Subjects

The dogs used were mesaticephalic and untrained other than for the experiments. They were privately owned pets and put to our disposal without economic compensation. The owners were informed about the nature of the experiments, asked about possible allergies or other food incompatibilities, and provided informed consent for their dogs to be used in the study. In the training, we exclusively used positive reinforcement, rewarding the dogs with food (Frolic, Mars Inc., McLean, USA) and praise. All animals were healthy and remained healthy for the duration of the experiments. We used three adult dogs of different sizes (9, 18, 40 kg) (Table S1).

#### Ethical statement

The experiments were approved by the Malmö/Lund ethical committee (permit M 148-12). All experiments were carried out in accordance with relevant guidelines and regulations. Sweden adopted the EU rules for research involving vertebrate animals in 2013 and under these regulations, our work with the dogs is considered normal handling and observation of domestic animals.

#### Experimental set-up

Training and testing was performed in a 2.3 ×3.4 m, temperature-controlled room in Biology Building B of Lund University. Ambient temperature (18.8–19.3 °C) was monitored with a digital thermometer (EN 13485, TFA Dostmann, Wertheim, Germany). The temperature of the dog’s rhinarium was measured with a thermographic camera (FLIR E30) before, during, and after each session. Biological tissue has high thermal emissivity (approx. 0.98, e.g.^49^ and ambient temperature was close to the skin’s temperature, such that reflected temperature was of minor importance. The emissivity setting of the camera was therefore kept at 1.0 because the possible error was minimal (max. 0.1 °C) and with this setting, the camera could also be used for measuring and checking the radiating temperature of the stimulus.

The room contained an experimental arena delimited on the long sides by sheets of dark plywood and on one short side by a wooden frame with a roller blind. A 15 W fan (Faset, Rusta, Upplands Väsby, Sweden) was blowing from the top of the frame 45° downward and towards the other short side of the arena where the stimuli were presented. While the dog was waiting outside the arena for the next trial, the blind was closed, preventing the dog from entering the arena and seeing how the next trial was set up (Fig. S2). A plywood divider, 1.6 m in length measured from the stimulus surfaces, separated the left and right stimuli. There was another 0.4 m between the end of the divider and the blind. The materials used in the set-up were of the same types and ages on both sides.

#### Thermal stimuli

The stimuli were generated with two 42 mm thick panels, 300 ×320 mm in surface area (Fig. S1). One face of each panel consisted of a 6 mm aluminum plate, carrying a heating wire driven by a low-voltage DC on the inside of the panel. The aluminum plate was connected with the other side, consisting of 12 mm plywood, by a hardwood frame equipped with a wooden handle. The frame was filled with 24 mm of expanded polystyrene foam to insulate the cold surface from the warm one. Both outer surfaces (aluminum and plywood) were covered with matt black adhesive plastic foil of high thermal emissivity (d-c-fix, Konrad Hornschuch, Weissbach, Germany). The driving voltage was adjusted such that the radiating temperature of the *warm* surface was about 11–13 °C above ambient temperature (31 ± 1 °C) to approximately match the stimulus to sources of thermal radiation relevant for a predator e.g.^47^. Despite insulation, some heat leaked over to the cold surface (the *neutral* stimulus), which was 1–2 °C warmer than ambient temperature. The voltage was turned on for both panels at least 30 min before a session in order to reach operating temperature and stayed on during the session to avoid acoustic cues caused by thermal movements. The fan was turned on and stayed on simultaneously.

The panels were positioned on sliding drawer mounts and held by magnets (Fig. S2). Each panel could be turned around by 180 degrees on its slider to present the *warm* surface on one side of the presenting apparatus and on the other side the *neutral* surface to the dog (Fig. S2), which means that there was always a *warm* and a *neutral* panel surface on both sides of the presenting apparatus. The panels were lifted off and put back on the sliders every time, even if they were not turned around, to avoid giving the waiting dog any acoustic or timing cues. Under both panels, shielded from any thermal radiation, there were bowls containing the same amount and type of food. Measurements with a thermal camera confirmed that the food remained at ambient temperature even during prolonged operation. On the *neutral* stimulus side, the sliding mechanism was blocked, invisibly to the dog, so that the food reward was inaccessible. On the *warm* stimulus side, the dog could push the slider backwards to access the food, during initial training touching the surface with its rhinarium so that the warm stimulus and the food reward were intuitively connected. Inequalities between the sides were avoided as much as possible by using materials of the same type and age, the same type and amount of food, the same, closed positions of the sliders, and the fan blowing from the dog’s position towards the presenting apparatus. This was intended to make it easy for the dogs to identify the stimuli of thermal radiation as the relevant stimuli. Remaining inequalities between the two sides in the set-up could not help the dogs to make correct choices because the animals equally often had to choose the left or the right side, while nothing was physically moved from left to right or vice versa. Stimuli were presented following computer-generated pseudo-random lists with a maximum of three consecutive equal choices in a row^50^. Longer series of equal choices may lead to side preferences of the animal, which would compromise the experiments. The centers of the panels were 460 mm apart (=16 degrees from leading edge of the divider).

#### Experimental procedure

The first steps in the training were to teach the dog the operation of the sliders and to make it realize that there was an accessible food reward on the warm side. The experimenter stood behind the sliders, with the radiating body heat shielded by plywood to a height of 1.4 m (Fig. S2). From this position, the experimenter opened the roller blind and called the dog into the arena. In the beginning, the slider with the *warm* stimulus was opened partially, so that the food reward became visible to the dog as soon as it had entered the arena. In addition, the experimenter pointed toward the *warm* side. When the dog had learned the basic procedure, the slider displaying the *warm* stimulus was also closed when the blind was opened and the experimenter pointed to the *warm* side with a small delay to let the dog collect sensory information before help was offered. Pointing was terminated when the focus of the entering dog had shifted from the experimenter toward the panels. A choice was recorded as correct or incorrect as soon as the dog’s head had passed the leading edge of the divider. Meaningful learning curves were not obtained because the dogs had help while learning the task.

When the dog without help consistently chose correctly in 70% of the training trials, stimulus size was reduced by covers that were hooked onto both panel surfaces facing the dog. The covers consisted of 10 mm expanded polystyrene foam laminated on 5 mm of Masonite and were painted black. Each cover concealed the entire panel surface facing the dog, except for a central hole 102 mm in diameter that let the radiation from the panel surface reach the dog. A free space of about 10 mm between the panel surface and the back of the cover allowed for undisturbed convection of air at the *warm* surface in order to avoid excessive warming of the panel. The covers reduced the stimulus to a solid angle of 3.7 degrees. Three covers were available, so that the one used on the *warm* panel surface could cool down before it was used again in order to avoid notable warming by continuous use. Rotating use of three covers also avoided any meaningful cues from nose prints left by the dog when pushing the *warm* side open.

Pointing was temporarily reintroduced (for several sessions) under these circumstances: after introduction of the covers, after prolonged periods of experimental inactivity (e.g. summer break), or if the dog focused only on the experimenter. For data collection, we did a maximum of 15 trials in each session, so that the dog could stay alert during the entire experiment. For each dog, there was an individually predefined stop criterion (Kevin: needed longer than 13 sec to make a choice two times in a row; Delfi: rhinarium temperature exceeded 21.5 °C; Charlie: needed longer than 13 seconds). Data collection sessions were at least five trials long and were performed double-blind. The experimenter left the room while a second person set up the trial. The experimenter entered the room and took the usual position, opened the blind, and without knowledge of the correct answer, called the dog into the arena. A few training sessions were necessary to let the dog accept the change in routine and reach the learning criterion again.

Double-blind testing took place only if rhinarium skin surface temperatures were 21.5 °C (M = 18.9, SD = 0.6) or lower. This is the upper limit of rhinarium temperatures observed in awake and alert dogs at 19 °C ambient temperature and considerably lower than in sleeping dogs^5^. We wanted to make sure that the dogs were ready to collaborate and testing sessions were therefore terminated if warming to temperatures higher than the above-mentioned limit occurred. Each data collection session consisted of at least five trials. The total number of testing sessions done with each dog depended on our access to the dog, its motivation, and rhinarium temperature dynamics.

#### Statistical analysis

The results from the double-blind trials were compared with the one-tailed cumulative binomial distribution to determine whether the dogs’ performances differed from chance level. The statistical tests were done using R Core Team, 2016.

### fMRI experiment

#### Subjects

Thirteen pet dogs, living with their owners, were tested (5 golden retrievers, 4 border collies, 1 Australian shepherd and 1 Chinese crested and 2 mixed breeds; aged 1.5–10 years (mean = 6.83, SD = 1.83); 5 females and 8 males) (Table S1). The owners of the dogs volunteered to participate in the training and testing procedure with their dogs, gave written informed consent and received no monetary compensation.

#### Ethical statement

Experimental procedures met the national and European guidelines for animal care and were approved by the local ethical committee (Állatkísérleti Tudományos Etikai Tanács KA-1719, Budapest, Hungary; Pest Megyei Kormányhivatal Élelmiszerlánc-Biztonsági és Állategészségügyi Igazgatósága XIV-I-001/520-4/2012, Budapest, Hungary).

#### Experimental set-up

The fMRI experiments took place at the MR Research Centre of the Semmelweis University Budapest, Hungary. The dogs were awake during the experiments and were trained to lie flat and motionless in the MR scanner. The training procedure (developed by Márta Gácsi^27^) preparing the dogs for awake fMRI experiments was based on positive reinforcement and social learning. Dogs were not restricted in any way and they could leave the scanner any time.

Adjacent to the MR scanner’s room was the operating and waiting room accommodating the computers and providing an area where dogs and all other human participants (dog owner, operator: controlling the scanner, experimenter: controlling the stimulus presentation, trainer: the dog’s MR trainer) could wait in between experimental runs.

The ambient temperature of the scanning room was set by a thermostat and was on average 22.5 °C (SD = 1.25 °C). The *warm* stimulus was on average 10.7 °C (SD = 0.95 °C) warmer than the ambient temperature.

In order to prepare the dogs for the specific circumstances of the study (e.g. people present at the scanner, apparatus used in the study), the dogs received 5–10 minute-long pre-conditionings before the measurements in the scanner’s waiting room and in the scanner. The experiment started cca. 5 minutes after the pre-conditioning phase (for details of the experimental procedure, see Supplementary Materials/fMRI Experimental procedure).

#### Thermal stimuli

Two types of stimuli were used in the experiment, presented by a ‘stimulus-presenting’ device (Fig. S3). A 60 × 100 mm *warm* surface and a 60 × 100 mm *neutral* (at ambient temperature) surface, both identically black, presented at 240 mm in front of the dogs’ nose. This distance was attained by making the dog position its nose at the end of a paper ruler, attached to the bottom of the ‘stimulus-presenting’ device (Fig. S3).

During the experiment, the ‘stimulus-presenting’ device was inside the scanner in front of the dog and was operated by the experimenter. The device was a 530 × 160 × 110 mm wooden frame box, enveloped by 20 mm thick layers of expanded polystyrene foam. Inside the insulating layers, there was a 60 × 100 × 400 mm glass cuboid, filled with warm (adjusted to the ambient temperature) water. One, 60 × 100 mm surface of the glass cuboid was covered in black electric tape and served as the *warm* stimulus (Fig. S3). The black electric tape’s high emissivity value made it suitable as the surface material. The device was equipped with two doors on the dog-facing end. The doors were operated by strings at the other end of the device, by the experimenter. The outer door (closer to the dog’s nose) presented the stimuli in each trial, making the *warm* or *neutral* stimulus visible upon opening. The inner door (farther from the dog’s nose) was insulated by a 20 mm thick layer of expanded polystyrene foam and was located right in front of the glass cuboid (Fig. S3). It was used to switch between the *warm* and *neutral* stimuli between trials. By closing or leaving it open, the experimenter could either cover (*neutral* stimulus) or leave the warm surface exposed (*warm* stimulus) upon opening the outer door. The dogs could not see the movement of this door, since it was only moved between trials when the outer door was closed. Importantly, the surface of the inner door facing the dog and the *warm* stimulus was covered with the same black electric tape, so the *warm* and *neutral* stimuli had essentially identical visual appearances.

#### Experimental procedure

The experiment consisted of 3, 5.5 minute long runs. At least 10–15 minute long breaks were kept between consecutive runs.

According to the two stimuli, there were two conditions: *warm* and *neutral*, presented in a block design. The presentation of the stimulus blocks started simultaneously with the measurement. The blocks were 2 × 2 second long displays of the stimuli, with a short break - closing and opening the outer door - in between. The intermittent presentation of the stimuli represented the presumably fluctuating perceptibility of naturally occurring warm stimuli. There were a total of 14 blocks in one run, with equal numbers of *warm* and *neutral* conditions. The blocks followed each other in a semi-random order (no more than two consecutive trials on the same side; first two trials on different sides), different in each of the 3 runs (3 different randomizations: rnd1, rnd2, rnd3). Blocks were separated by baseline periods of varying length (7–10 seconds, on average: 8.5 seconds). The order in which the dogs participated in the 3 runs was balanced to the extent possible for 13 subjects (2 dogs/5 permutations, 3 dogs/1 permutation). (For data acquisition details, see Supplementary Materials/fMRI experiment-Data acquisition).

#### Data analysis

For the preprocessing and analyses of the images we used MATLAB R2016b (http://www.mathworks.com/products/matlab/) and SPM12 (http://www.fil.ion.ucl.ac.uk/spm)^51^. Preprocessing consisted of the following steps. The functional EPI-BOLD images were first realigned. The average of maximal movements per dog was below 1.5 mm for the translation directions, and below 0.01 radians for the rotation directions. The anatomical images of the dogs were then transformed into a common space, with a selected template (golden retriever, male, 7.5 years), using the Thermo Scientific Amira for LifeSciences 6.0 software platform (https://www.thermofisher.com/us/en/home/industrial/electron-microscopy/electron-microscopy-instruments-workflow-solutions/3d-visualization-analysis-software/amira-life-sciences-biomedical.html). The mean functional image was registered to the now normalized anatomical image, using the Amira software, resulting in a normalized mean functional image. The transformation matrix between the mean functional image and the normalized mean functional image was estimated by SPM’s standard nonlinear warping function with 16 iterations and the space was centered around the commissura rostralis, as origo^52^, analogously to the MNI coordinate system used in humans^53^. The resulting transformation matrix was then applied to all realigned functional images. Finally, for spatial filtering, normalized functionals were convolved with an isotropic 3-D Gaussian kernel (FWHM = 4 mm).

The analysis of the fMRI data was performed using a general linear model and statistical parametric mapping. One model was constructed with condition regressors for each run and for both block types: *warm* and *neutral*. Conditions were modeled as 2 second long blocks. To model potential motion artifacts, realignment regressors for each run were also included. To remove low-frequency signals, a high-pass filter with a cycle-cutoff of 128 second was used. Regressors were convolved with the canonical haemodynamic response function of SPM. We tested one t-contrast in our single-subject fixed effect analyses: *warm* vs. *neutral* stimulus (W > N). On the group level, the contrast images generated for individual subjects were entered into a one sample random effects analysis model. An overall voxel threshold of p < 0.001 was applied, and only clusters FWE-corrected for multiple comparisons (on the cluster level) were considered as significant effects (p < 0.05).

To assess hemispheric lateralization effects, we compared the percent signal change of the *warm* > *neutral* contrast observed in a 4mm-radius spherical volume around the peak voxel (x = −12, y = −14, z = 18, r = 4 mm)^52^ and its counterpart in the right hemisphere (x = 12, y = −14, z = 18, r = 4 mm)^52^ in a Mann-Whitney-U test. The average parameter estimates were calculated within that volume, using the subject specific beta images as input. The percent signal changes were calculated for each subject based on the average beta values of the selected sphere (toolbox: WFU_pickatlas 3.0.5 (http://fmri.wfubmc.edu/software/pickatlas). All statistical analyses were performed using IBM SPSS 22 (https://www.ibm.com/products/spss-statistics).

## Supplementary information

Supplementary Information.

## Data Availability

All data are available upon request, which requests should be addressed to: nani.balint@gmail.com. Informed consent has been obtained from Alix Brusseau to publish the image (Fig. S2) in an online open-access publication.

## References

[1] Hill B (1948). Rhinoglyphics: Epithelial sculpture of the mammalian rhinarium. J. Zool. (London).

[2] Catania KC (2000). Epidermal sensory organs of moles, shrew-moles, and desmans: A study of the family Talpidae with comments on the function and evolution of Eimer’s organ. Brain Behav. Evol..

[3] Barker DJ, Welker WI (1969). Receptive fields of first-order somatic sensory neurons innervating rhinarium in coati and raccoon. Brain Res..

[4] Gläser N, Kröger RHH (2017). Variation in rhinarium temperature indicates sensory specializations in placental mammals. J. Therm. Biol..

[5] Kröger RHH, Goiricelaya AB (2017). Rhinarium temperature dynamics in domestic dogs. J. Therm. Biol..

[6] Evans, H. E. & de Lahunta, A. *Miller’s Anatomy of the Dog*., a: chapter 9, b: chapter 18, c: chapter 3 (Elsevier, Amsterdam, 2013).

[7] Gescheider GA, Thorpe JM, Goodarz J, Bolanowski SJ (1997). The effects of skin temperature on the detection and discrimination of tactile stimulation. Somatosens. Mot. Res..

[8] Cadena V, Andrade DV, Bovo RP, Tattersall GJ (2013). Evaporative respiratory cooling augments pit organ thermal detection in rattlesnakes. J. Comp. Physiol. A Sens. Neural Behav. Physiol..

[9] Bakken GS, Schraft HA, Cattell RW, Tiu DB, Clark RW (2018). Cooler snakes respond more strongly to infrared stimuli, but we have no idea why. J. Exp. Biol..

[10] Newbold HG, King CM (2009). Can a predator see ‘invisible’ light? Infrared vision in ferrets (*Mustela furo*). Wildl. Res..

[11] Ala-Laurila P, Pahlberg J, Koskelainen A, Donner K (2004). On the relation between the photoactivation energy and the absorbance spectrum of visual pigment. Vision Res..

[12] Campbell AL, Naik RR, Sowards L, Stone MO (2002). Biological infrared imaging and sensing. Micron.

[13] Bullock TH, Diecke FPJ (1956). Properties of an infra‐red receptor. J. Physiol. (Cambrigde).

[14] Bakken GS, Colayori SE, Duong T (2012). Analytical methods for the geometric optics of thermal vision illustrated with four species of pitvipers. J. Exp. Biol..

[15] Gracheva EO (2010). Molecular basis of infrared detection by snakes. Nature.

[16] Kardong KV, Mackessy SP (1991). The strike behavior of a congenitally blind rattlesnake. J. Herpetol..

[17] Grace MS, Woodward OM, Church DR, Calisch G (2001). Prey targeting by the infrared-imaging snake *Python molurus*: Effects of experimental and congenital visual deprivation. Behav. Brain Res..

[18] Nowak, R. M. Walker’s Mammals of the World. 6th edition, vol. 1, (John Hopkins University Press, Baltimore, ed. 6, pp. 665 1999).

[19] Uemura, E. E. Fundamentals of Canine Neuroanatomy and Neurophysiology (John Wiley & Sons, 2015).

[20] Nickel, R., Schummer, A. & Seiferle, E. *Lehrbuch der Anatomie der Haustiere, Band IV: Nervensystem, Sinnesorgane, Endokrine Drüsen*. *4., Unveränderte Auflage* (Berlin u.a.: Enke 2003).

[21] Hammer DX, Schmitz H, Schmitz A, Rylander A, Welch AJ (2001). Sensitivity threshold and response characteristics of infrared detection in the beetle *Melanophila acuminata* (Coleoptera: Buprestidae). Comp. Biochem. Physiol. Part A Mol. Integr. Physiol..

[22] Goris RC (2011). Infrared organs of snakes: An integral part of vision. J. Herpetol..

[23] Kürten L, Schmidt U (1982). Thermoperception in the common vampire bat (*Desmodus rotundus*). J. Comp. Physiol..

[24] Whitlock JR, Sutherland RJ, Witter MP, Moser MB, Moser EI (2008). Navigating from hippocampus to parietal cortex. Proceedings of the National Academy of Sciences.

[25] Jia H (2014). Functional MRI of the olfactory system in conscious dogs. Plos One.

[26] Jia H (2015). Enhancement of odor-induced activity in the canine brain by zinc nanoparticles: A functional MRI study in fully unrestrained conscious dogs. Chemical Senses.

[27] Andics A, Gácsi M, Faragó T, Kis A, Miklósi Á (2014). Voice-sensitive regions in the dog and human brain are revealed by comparative fMRI. Current Biology.

[28] Andics A (2016). Neural mechanisms for lexical processing in dogs. Science.

[29] Leliveld LM, Langbein J, Puppe B (2013). The emergence of emotional lateralization: evidence in non-human vertebrates and implications for farm animals. Applied Animal Behaviour Science.

[30] Giljov AN, Karenina KA, Malashichev YB (2009). An eye for a worm: lateralisation of feeding behaviour in aquatic anamniotes. Laterality: Asymmetries of Body, Brain and Cognition.

[31] Robins A, Chen P, Beazley LD, Dunlop SA (2005). Lateralized predatory responses in the ornate dragon lizard *(Ctenophorus ornatus)*. NeuroReport.

[32] Koboroff A, Kaplan G, Rogers LJ (2008). Hemispheric specialization in Australian magpies *(Gymnorhina tibicen)* shown as eye preferences during response to a predator. Brain Research Bulletin.

[33] Rogers LJ (2002). Lateralization in vertebrates: its early evolution, general pattern, and development. Advances in the Study of Behavior.

[34] Rogers, L.J., Vallortigara, G. & Andrew, R.J. *Divided brains: the biology and behaviour of brain asymmetries* (Cambridge University Press, 2013).

[35] Ocklenburg, S. & Gunturkun, O. *The lateralized brain: The neuroscience and evolution of hemispheric asymmetries* (Academic Press, 2017).

[36] Forrester, G., Hudry, K., Lindell, A. & Hopkins, W. D. *Cerebral lateralization and cognition: evolutionary and developmental investigations of behavioral biases* (Vol. 238, Academic Press, 2018).

[37] Siniscalchi M, Pergola G, Quaranta A (2013). Detour behaviour in attack trained dogs: left-turners perform better than right-turners. Laterality.

[38] Lee GP (2004). Neural substrates of emotion as revealed by functional magnetic resonance imaging. Cognitive and Behavioral Neurology.

[39] Racca, A., Guo, K., Meints, K. & Mills, D. Reading Faces: Differential Lateral Gaze Bias in Processing Canine and Human Facial Expressions in Dogs and 4-Year-Old Children. *PLoS One* **7**(4), p. e36076. (2012). 10.1371/journal.pone.0036076 PMC3338636 22558335

[40] Siniscalchi M, d’lngeo S, Quaranta A (2017). Lateralized Functions in the Dog Brain. Symmetry.

[41] Siniscalchi M, D’Ingeo S, Quaranta A (2016). The dog nose ‘KNOWS’ fear: Asymmetric nostril use during sniffing at canine and human emotional stimuli. Behavioural Brain Research.

[42] Quaranta A, Siniscalchi M, Vallortigara G (2007). Asymmetric tail-wagging responses by dogs to different emotive stimuli. Current Biology.

[43] Racca, A., Guo, K., Meints, K. & Mills, D. Reading Faces: Differential Lateral Gaze Bias in Processing Canine and Human Facial Expressions in Dogs and 4-Year-Old Children. *Plos One* **7**(4), p. e36076 (2012). 10.1371/journal.pone.0036076 PMC3338636 22558335

[44] Siniscalchi M, Quaranta A, Rogers L (2008). Hemispheric Specialization in Dogs for Processing Different Acoustic Stimuli. Plos One.

[45] Elofsson R, Kröger RHH (2018). A variation of pigmentation in the glabrous skin of dogs. J. Morphol..

[46] Amemiya F (1995). The surface architecture of snake infrared receptor organs. Biomed. Res. (Tokyo).

[47] Ebert J, Westhoff G (2006). Behavioural examination of the infrared sensitivity of rattlesnakes (*Crotalus atrox*). J. Comp. Physiol. A Sens. Neural. Behav. Physiol..

[48] Rogalski, A. *Infrared Detectors* (CRC press, Boca Raton, ed. 2, 2010), chapter 6 (2010).

[49] Martello LS, Da Luz SS, Gomes RC, Corte RSRP, Leme PR (2016). Infrared thermography as a tool to evaluate body surface temperature and its relationship with feed efficiency in *Bos indicus* cattle in tropical conditions. Int. J. Biometeorol..

[50] Gellermann LW (1933). Chance orders of alternating stimuli in visual discrimination experiments. The Pedagogical Seminary and Journal of Genetic Psychology.

[51] Penny, W. D., Friston, K. J., Ashburner, J. T., Kiebel, S. J. & Nichols, T. E. Eds., *Statistical Parametric Mapping: the Analysis of Functional Brain Images* (Elsevier, London, 2011).

[52] Czeibert, K., Andics, A., Petneházy, Ö. & Kubinyi, E. A detailed canine brain label map for neuroimaging analysis. *Biologia Futura* **70**(2), 112–120 (2019). 10.1556/019.70.2019.14 34554420

[53] Chau, W. & McIntosh, A. R. The Talairach coordinate of a point in the MNI space: how to interpret it. *Neuroimage* **25**(2), 408–416 (2005). 10.1016/j.neuroimage.2004.12.007 15784419

