# Supplementary material for: Dogs can sense weak thermal radiation
Source: Sci Rep. 2020 Feb 28;10:3736. doi: 10.1038/s41598-020-60439-y (PMC7048925; doi:10.1038/s41598-020-60439-y)
Supplement: Supplementary file 1 — Supplementary Information. [file 41598_2020_60439_MOESM1_ESM.pdf]

Supplementary information

Dogs can sense weak thermal radiation

Anna Bálint[1,2,3*], Attila Andics[3,4], Márta Gácsi[2,3], Anna Gábor[3,4], Kálmán Czeibert[3], Chelsey M. Luce[1,5], Ádám Miklósi[2,3], Ronald H.H Kröger[1]

fMRI Experimental procedure

Four people participated in the experiments, the operator (Op), the experimenter (E), the dog's 'MRI' trainer (T) and the dog's owner (O).

A. Pre-conditioning

The pre-conditioning consisted of two phases. The first one took place in the MR scanner's waiting room (adjacent to it), with the participation of E, T and O. E put the 'stimulus-presenting' device on the floor and the dog was let to inspect it. Then, the dog was asked to lie down in front of it, with its nose approximately at the end of the paper ruler. E started moving the doors, similarly to the experimental conditions for cca.3 minutes, while T and O encouraged the dog to lie still.

The second phase took place in the scanning room, with the assistance of E, T, O in the scanning room and Op in the computer room, controlling the scanner. The device was mounted on the scanner bed inside the scanner. The dog was lifted on the bed and was asked to put its head in front of the device, with the tip of its nose at the end of the paper ruler. After fixing the coil on the dog's head, the scanner bed was drawn inside the bore. E, T and O took up their assigned positions (see next section) in the scanning room, and the Op started a 1-minute long measurement session, without E presenting the stimuli. This way, the dog could familiarize with the exact experimental conditions before the measurements started.

After extensive praising and rewarding, the dog was lifted off the bed and all left the scanning room for a cca.5-minute break.

B. Experiment

During the break between the pre-conditioning phase and the measurements, E prepared the 'stimulus-presenting' device for the experiment by filling up the glass cuboid with warm tap water. The temperature of the water was adjusted to the temperature in the scanning room and was controlled by a thermographic camera.

Before and after each experimental run, E took thermographic measurements of the dogs' nose and the warm stimulus' temperature. Since these measurements could not be taken in the scanning room where the actual experiment took place due to technical reasons, they were taken in the waiting room. However, the difference between the ambient temperature of the waiting room and the scanning room was on average only 1.2 °C (SD=0.6°C). The temperature of the scanning room was measured by a built-in thermometer and was displayed in the computer room.

The device was mounted on the scanning bed again. The dog was lifted on the bed and was asked to put its nose in the required position. During the experiment (and pre-conditioning), E, T and O had assigned positions in the scanning room. E and O were at the front end of the scanner, E was sitting on a chair, invisible to the dog, while O was standing, looking at the dog throughout the experiment. T was standing at the far end of the scanner, having both the dog and the Op in the computer room in her line of sight. Since both O and T had a reliable view on the dog, they could detect and end the measurement in case the dog was moving.

E presented the stimuli by operating the device according to a pre-determined order.

Instructions on the order and the timing of door openings were recorded on an audio file and were played back to E on a headphone.

At the end of the test, the Op signaled T who then signaled the others in the scanning room. After extensive praising and rewarding, the dog was lifted off the bed and all left the scanning room.

fMRI experiment-Data acquisition

The MRI measurements were performed using a Philips Ingenia 3.0 T whole body MR system. We used a Philips SENSE Flex Medium coil. The EPI-BOLD fMRI time series were obtained from 30 transverse slices, covering the whole brain. The spatial resolution was 2×2×2 mm, with a 0.5 mm slice gap. A single-shot gradient-echo planar sequence was used (ascending slice order; acquisition matrix 96×57; TR = 3200 ms, including 2000 ms acquisition and 1200 ms silent gap; TE = 30 ms; flip angle = 90°). All runs included 75 volume acquisitions. As anatomical reference, a standard T1-weighted three-dimensional scan was collected (turbo-field echo TFE) sequence; 180 slices; 1×1×1 mm spatial resolution). Threshold of motion was set to 3 mm translation or more than 3° rotation in any direction. Runs exceeding the threshold were discarded and repeated. In case of unsuccessful runs, the dogs were placed in the scanner at a maximum of 3 times in a row.

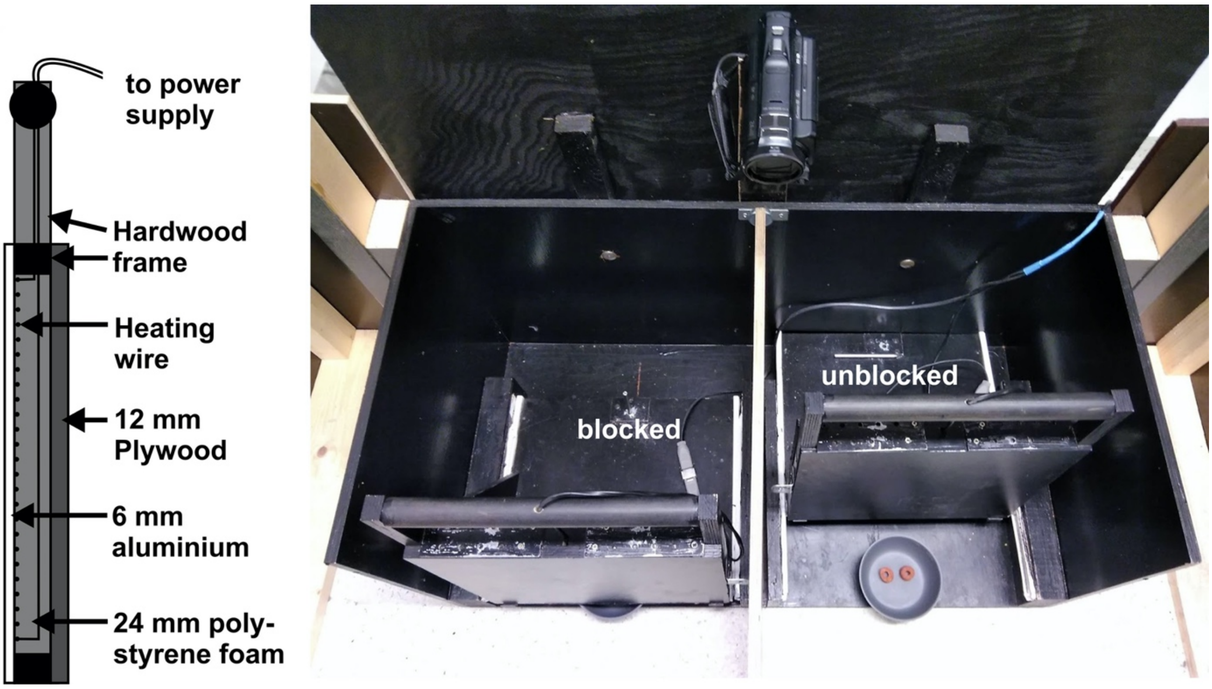

Figure S1. Device generating the stimuli in the behavioral experiment. a, Cross-section through a custom-crafted panel used to generate *warm* and *neutral* stimuli for behavioral training and testing. The heated or the insulated surface was turned toward the dog to generate a *warm* or *neutral*, respectively, stimulus. **b,** Photograph of the stimulation apparatus with two panels on sliding mounts. The *warm* side is pushed back, exposing the food reward underneath. There was an equivalent reward also under the *neutral* side, but the sliding mechanism was blocked, such that the dog could not get access. For changing the stimuli from *warm* to *neutral* and vice versa, the panels stayed in place, but were turned around, meaning that there always was a *warm* and a *neutral* surface on both sides of the stimulation apparatus. Both panels were in the forward positions (closed) when the dog made its choice (see Fig. S2a).

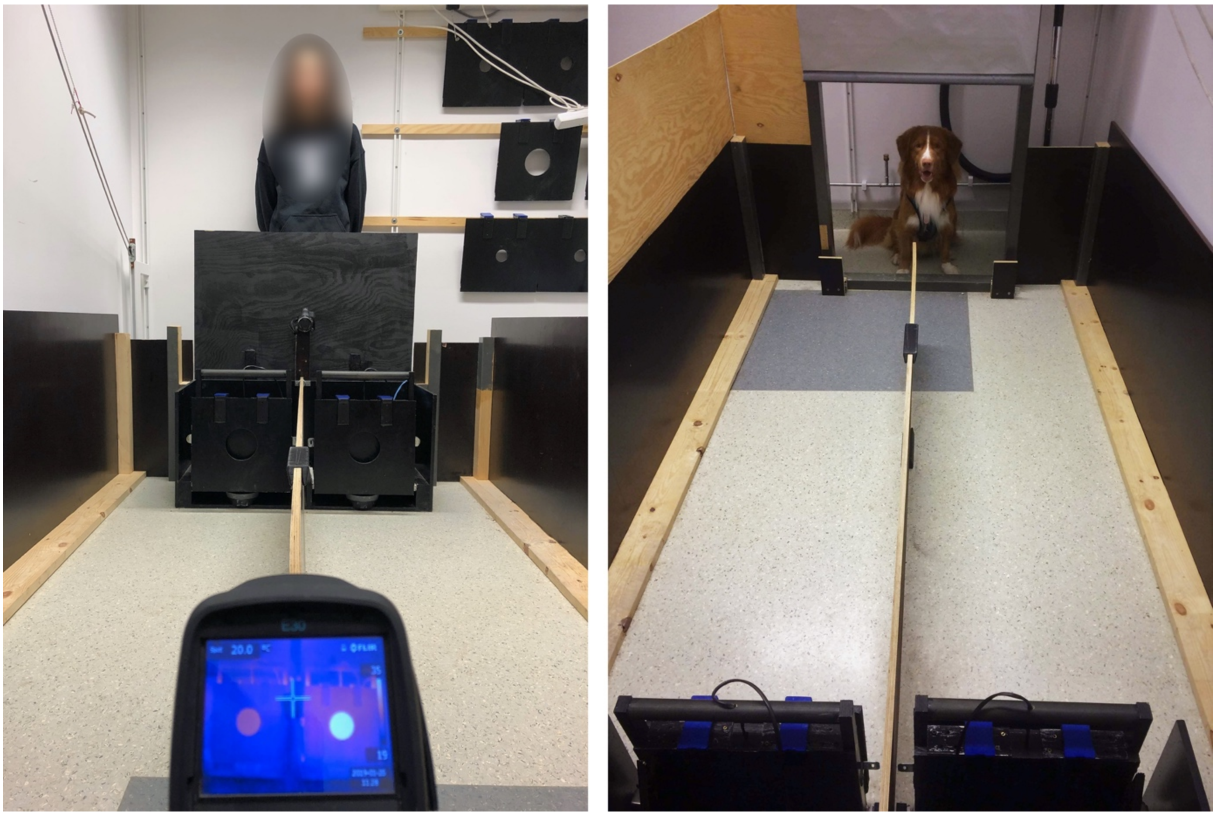

Figure S2. Experimental arena for the behavioral experiment. a, Dog's view (appearance in the IR is demonstrated on the screen of the E30 thermal camera). Both panels are in the forward positions and covers with central holes 102 mm in diameter are hooked on to make the task more difficult for the dog. Note the similar visual appearances of both sides, contrasted by the difference in the infrared, as shown on the screen of the thermal camera. On the left side in this image, the *neutral* surface of the panel faced the dog, while the *warm* surface had been turned toward the experimenter. The position of the blue cable supplying both panels with low voltage DC never changed and did therefore not indicate on which side the *warm* surface of the panel was facing the dog. **b,** Experimenter's view. The blind behind which the dog had to wait while a trial was set up is opened and the dog waits to be called into the arena. From the frame holding the roller blind, about 1 m above the head of the dog, a fan was blowing approx. 45 degrees downward toward the presenting apparatus. Note the divider that separates the *warm* and *neutral* sides, forcing the dog to make its choice from a distance of at least 1.6 m.

Informed consent has been obtained from Alix Brusseau to publish the image in an online open-access publication.

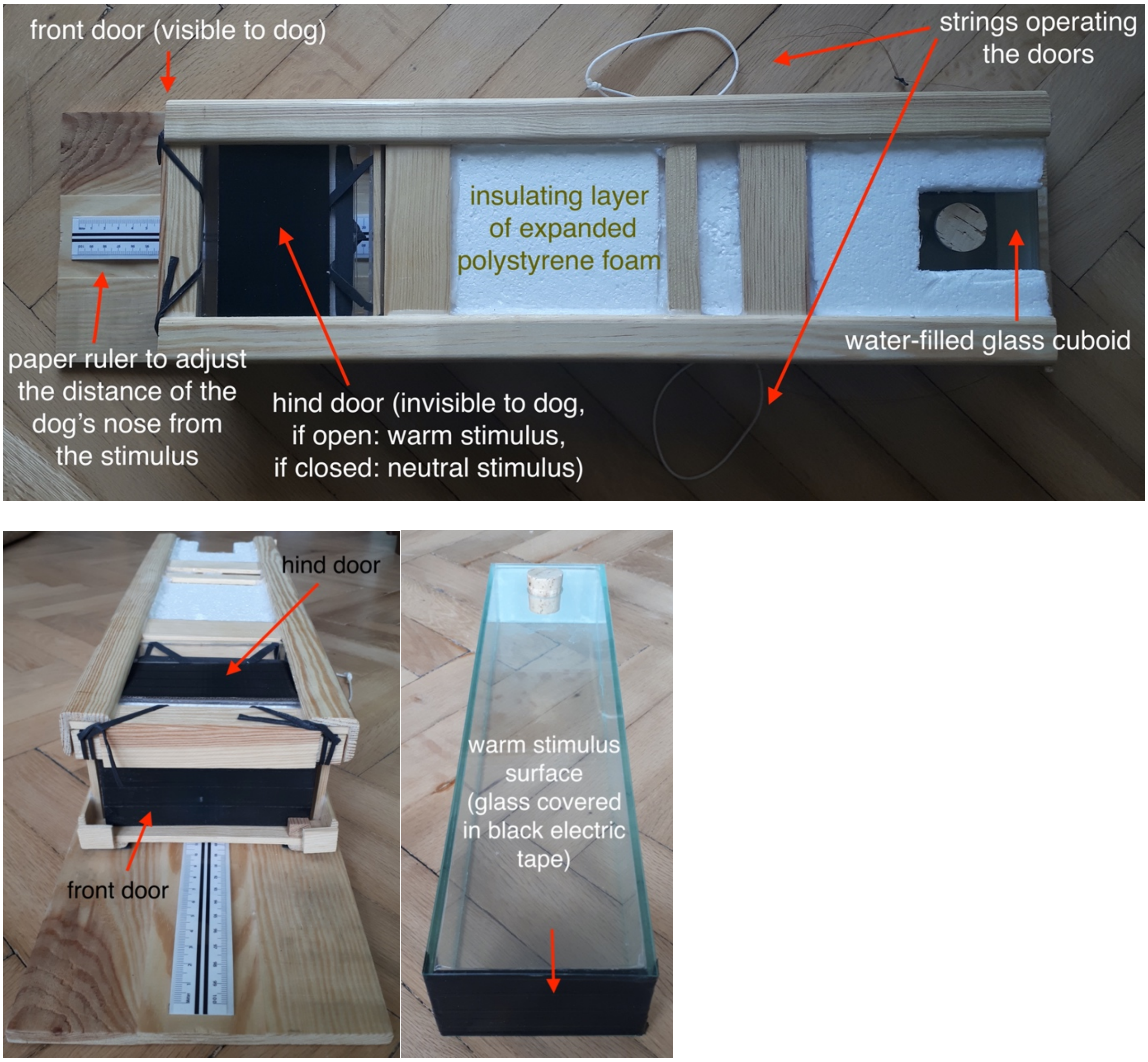

Figure S3. Stimulus-presenting device in the fMRI experiment. a, top view of the device **b,** top-front view of the device **c,** warm-water filled glass cuboid, with one surface covered in black electric tape, serving as the warm stimulus surface.

Behavioral experiment					
**Subject**	**Breed**	**Age (yrs)**	**Weight (kg)**	**Sex**	**Order of RND**
Kevin	golden retriever	9	40	male	-
Delfi	Nova Scotia duck tolling retriever	1.5	18	male	-
Charlie	mixed breed	4	9	female	-

fMRI experiment					
**Subject**	**Breed**	**Age (yrs)**	**Weight (kg)**	**Sex**	**Order of RND**
Maya	golden retriever	6	25	female	1-2-3
Apacs	chinese crested	2.5	10.8	male	1-3-2
Bran	border collie	6	17.5	female	3-2-1
Barney	golden retriever	7.5	40	male	3-1-2
Maverick	border collie	8	16	male	2-1-3
Akira	mixed breed	1.5	25	female	2-3-1
Bodza	golden retriever	2.5	26.5	female	1-2-3
Bingó	mixed breed	1.5	27	male	2-1-3
Barack	golden retriever	2.5	24.5	female	3-2-1
Grog	border collie	10	21	male	2-3-1
Pán	aussie	2.5	24.5	male	3-1-2
Monty	border collie	6	19	male	1-3-2
Sander	golden retriever	5	30	male	1-2-3

Table S1. Subjects of the behavioral and fMRI experiment. Order of RND: order of the 3 different randomizations.
